# Bach2: A Key Regulator in Th2-Related Immune Cells and Th2 Immune Response

**DOI:** 10.1155/2022/2814510

**Published:** 2022-03-12

**Authors:** Guo Liu, Feng Liu

**Affiliations:** Department of Otolaryngology-Head and Neck Surgery, West China Hospital of Sichuan University, Chengdu, China

## Abstract

Th2 immune response is essential for providing protection against pathogens and orchestrating humoral immunity. However, excessive Th2 immune response leads to the pathogenesis of Th2 inflammation diseases, including asthma, allergic rhinitis, and atopic dermatitis. Emerging evidence suggest a critical role of the transcription factor Bach2 in regulating Th2 immune responses. Bach2 serves as a super enhancer and transcriptional repressor to control the differentiation and maturation of Th2-related immune cells such as B cell lineages and T cell lineages. In B cells, Bach2 is required for every stage of B cell development and can delay the class switch recombination and antibody-producing plasma cell differentiation. In T cell lineages, Bach2 suppresses the CD4^+^ T cell differentiation into Th2 cells, restrains Th2 cytokine production, and promotes the generation and function of regulatory T (Treg) cells to balance the immune activity. Furthermore, studies in various animal models show that Bach2 knockout animals spontaneously develop Th2 inflammation in the airway and gastrointestinal tract. Genome-wide association studies have identified various susceptibility loci of Bach2 which are linked with Th2 inflammatory diseases such as asthma and inflammatory bowel disease. Here, we discuss the critical role of Bach2 involved in the Th2 immune response and associated inflammatory diseases.

## 1. Introduction

T helper (Th) 2 immune response, which belongs to type 2 immune response, represents the typical adaptive response against various allergens or extracellular parasitic infections. Through inducing Th2 cytokine release, immunoglobulin (Ig) E antibody production, and inflammatory mediator secretion, Th2 immune response provides adequate protection against these infections [1]. However, the unbalanced adaptive immune responses excessively driven towards Th2 immunity due to constant antigen exposure could lead to Th2 inflammation diseases, including asthma, allergic rhinitis, and atopic dermatitis [2, 3].

In recent years, studies have discovered that the transcription factor BTB and CNC homologue 2 (Bach2) play a crucial role in regulating Th2 immune responses. The high expression of Bach2 in T cells and B cells suggests that it has a key role in these cells [4]. Bach2 deficiency in CD4^+^ T cells reduces the number of naïve CD4^+^ T cells and regulatory T (Treg) cells and increases the number of Th2-type effector memory T cells, indicating that Bach2 is closely related to Th2 cell-mediated immune responses and Treg cell-mediated immune homeostasis [4–6]. In addition, early studies have found that Bach2 also plays an important role in humoral immunity by promoting B cell differentiation and affecting high-affinity antibody production [7, 8]. Recent studies have further demonstrated that Bach2 has a critical role in controlling Th2 inflammation, because Bach2 knockout animals spontaneously develop Th2-mediated inflammation in the lung and small intestine [9, 10]. In this review, we discuss the differential roles of Bach2 involved in Th2 immune responses and Th2 inflammation.

## 2. Bach2 Basics

**Figure 1:**
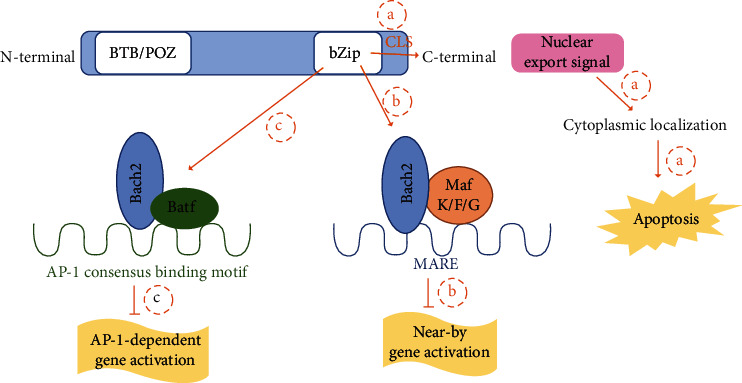
Schematic representation of Bach2 protein structure and its associated functions. (a) Cytoplasmic localization signal (CLS) in bZip region leads to Bach2 cytoplasmic localization and subsequently cell apoptosis. (b) Bach2, via the bZip region, forms heterodimer with small Maf protein, binds to Maf-recognition elements (MARE), and inhibits near-by gene activation. (c) Bach2, via the bZip region, forms Bach2/Batf complex, binds to AP-1 consensus binding motif, and inhibits AP-1-dependent gene activation.

Bach2 is a member of the Bach family which belongs to the BTB-basic leucine zipper (bZip) transcription factor family [7]. The Bach2 gene is located on human chromosome 6 (6q15) and mouse chromosome 4 (4A4), which encodes a 741 amino acid protein with highly conserved functional domains [4]. Bach2 possesses both BTB/POZ and bZip DNA-binding domains, which can either bind directly to DNA or dimerizes with other transcription factors via protein-to-protein interactions. The BTB/POZ domain is located at the N-terminus of the Bach2 protein, while the bZip domain is located at the C-terminus and contains a cytoplasmic localization signal (CLS) [4, 7]. The CLS controls the subcellular localization of the Bach2 protein by cytoplasmic accumulation of Bach2 through its C-terminal nuclear export signal. Under an oxidative stress condition, CLS induces Bach2 protein accumulation in the nucleus and subsequently apoptosis [7] (Figure 1).

The bZip region is required for Bach2 to form heterodimers with small Maf proteins, including MafK, MafF, and MafG [4, 7]. A complex of Bach2 and small Maf proteins binds to Maf-recognition elements (MAREs) and recognizes the consensus DNA sequence (TGCTGA(G/C)TCAGCA) to suppress the expression of downstream genes [4, 7, 11]. Hence, the formation of Bach2/small Maf protein heterodimers plays a critical role in Bach2-mediated transcription repression. Moreover, Bach2 binds to the basic leucine zipper transcription factor ATF-like (Batf) through its bZip region to form a Bach2/Batf complex. The Batf family belongs to the AP-1 family of transcription factors and is required for Th-cell subset differentiation [12, 13]. Once the Bach2/Batf complex is formed, it binds to the AP-1 consensus motif and suppresses AP-1-dependent gene activation [6] (Figure 1).

## 3. Bach2 and Th2 Response-Related Immune Cells

Th2 immune response is characterized by the generation of Th2 cells, production of IgE antibody, and activation of eosinophil and mast cells [14]. After initial antigen exposure, professional antigen-presenting cells, such as dendritic cells (DCs), present antigens to naïve CD4^+^ T cells, differentiating the majority of these cells into Th2 cells and some to follicular T helper (Tfh) cells. Then, Th2 and Tfh cells orchestrate a Th2 immune response through the production of Th2 cytokines, such as interleukin-4 (IL-4), IL-5, and IL-13 [15, 16]. IL-4 secreted from Th2 and Tfh cells can initiate B cell class switching to IgE and IgG1 production [15]. IgE binds to its high-affinity receptors on basophils and mast cells, leading to their activation and secretion of several cytokines and inflammatory mediators such as histamine, heparin, and serotonin. These factors subsequently prompt effector function characteristics of Th2 inflammation, including the recruitment of alternatively activated macrophages and granulocytes, inducing smooth muscle contractility, and causing mucus hypersecretion [14].

Th2 immune response is a complex process involving cellular immunity, humoral immunity, and immune tolerance. Th2 cells are considered to be the main effector cells of cellular immunity, while B cells and Treg cells are chiefly responsible for humoral immunity and immune tolerance [14]. Bach2 was initially discovered in B cells and later identified in all T-cell lineages [7, 11, 17]. Evidence shows that Bach2 plays a critical role in regulating the differentiation of these Th2-related immune cells (including Th2 cells, B cells, Tfh cells, and Treg cells) at the transcriptional level, thereby restraining Th2 cytokine production, high-affinity antibody generation, and suppressing excessive immune activation [5, 6, 10, 18, 19]. In this section, we discuss the role of Bach2 in the transcriptional regulation network of Th2-related immune cells.

### 3.1. The Effect of Bach2 on Th2 Cell Differentiation and Function

Bach2 is deemed as a negative regulator in CD4^+^ T cells. It can suppress effector memory-related genes to maintain CD4^+^ T cells in a naïve state [20]. The research by Tsukumo et al. [20] and Kuwahara et al. [6] found that Bach2 deficiency results in the reduction of naïve CD4^+^ T cells and enhances effector Th-cell differentiation, particularly Th2 cells. Bach2-deficient CD4^+^ T cells were highly expressed with Th2-related genes such as *gata3*, *il4*, and *Prdm1* (encodes the Blimp-1) after T-cell receptor (TCR) stimulation and produced more Th2 cytokines (IL-4, IL-5, and IL-13) than those derived from normal CD4^+^ T cells under the IL-2 plus IL-4 culture conditions [6, 20]. Furthermore, the upregulation of Th2 cytokines was also observed even when the Bach2-deficient CD4^+^ T cells were cultured in the presence of IL-12 (the cytokine that promotes Th1 cell differentiation), suggesting that Bach2-deficient CD4^+^ T cells are strongly predisposed to differentiate into Th2 cells [20]. On the other hand, Kuwahara et al. constructed a Bach2-overexpressed mice model to upregulate the Bach2 mRNA expression by 2.5-fold. However, the development of Foxp3^+^ CD4^+^ T cells was not changed in these mice, accompanied by reduced IL-4-producing CD4^+^ T cells and Th2 cytokines [6]. This indicates that Bach2 suppresses Th2 cell differentiation independent of Treg-mediated regulation. Moreover, the H3K27 acetylation and histone H3K4 tri-methylation at the Th2 cytokine gene loci are not decreased in Bach2-deficient Th2 cells, suggesting that Bach2 suppresses Th2 cell differentiation without repressing the chromatin remodeling [6].

**Figure 2:**
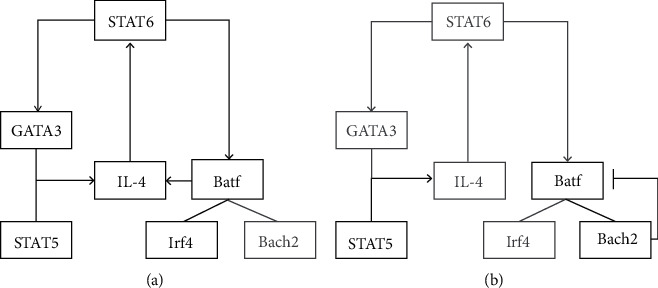
The core transcription factor regulatory network of Th2 cell differentiation. (a) Two positive feedback loops, (i) IL-4/STAT6/GATA3 and (ii) IL-4/STAT6/Batf–Irf4, promote the complete differentiation of Th2 cells. (b) Bach2 replaces Irf4 to form an inhibiting Batf–Bach2 dimer, which interferes with the IL-4/Batf–Irf4 amplification loop to restrain excessive Th2 cell differentiation. Black boxes and arrows represent activated genes and pathways, and gray represent surpressed genes and pathways.

Bach2 may inhibit Th2 cell differentiation through two pathways, according to the research by Kuwahara et al. [6]. First, Bach2 forms a transcription complex with Batf to interfere with the IL-4/Batf–Irf4 amplification loop to restrain excessive Th2 cell differentiation. It is well known that differentiation of naïve CD4^+^ T cells into effector Th2 cells requires two positive feedback loops in the transcriptional regulation network: (1) IL-4/STAT6/GATA3 loop and (2) IL-4/Batf–Irf4 loop [3, 21, 22] (Figure 2(a)). In the IL-4/Batf–Irf4 loop, Bach2 replaces the Irf4 to form a negative transcriptional complex with Batf. Then, the Bach2–Batf complex competes with Batf–Irf4 for binding to the AP-1 motif in the regulatory region of the Th2 cytokine locus and inhibits the production of Th2 cytokines, such as IL-4 [6]. Second, the Bach2–Batf complex can directly bind to the Batf and Batf3 gene loci to suppress their transcription, thereby reducing the formation of Batf-related complex (such as the Batf–Irf4) [6]. Obviously, there is a regulator network of Th2 cell differentiation among IL-4, Bach2–Batf, and Batf–Irf4, in which Bach2 plays as a key negative regulator to control Th2 differentiation and Th2 immune response (Figure 2(b)).

### 3.2. The Effect of Bach2 on B Cell Differentiation and Function

Bach2 is detected at every developmental stage of B cells except in plasma cells [8, 23]. At the pro-B cell stage, Bach2 has been postulated to determine the lineage selection of B cells in progenitor cells. It promotes B cell commitment by directly repressing the myeloid-related genes, such as *Cebpa* and *Cebpb* [24, 25].

From pro-B cells to pre-B cells, paired box protein 5 (Pax5), the master regulator of B cell differentiation, and the Rag recombinases are activated. The upregulated expression triggers the gene rearrangement of the immunoglobulin heavy chain (*V*_*H*_–DJ_*H*_ recombination) [26]. Meanwhile, Pax5 activates the expression of Bach2 before the heavy chain checkpoint and continuously increases B cell lymphoma 6 (Bcl6) levels [27]. Then, Bach2 serves as a crucial mediator to execute the negative selection of early B cells with failed *V*_*H*_–DJ_*H*_ rearrangements by binding to the promoter of *ARF/TP53* to induce the p53 expression, which in turn mediates a program of cell death [28, 29]. In contrast, Bcl6 instead of Bach2 suppresses the p53 expression also by binding to *ARF/TP53* and mediates the positive selection of pre-B cells with successful rearrangements to pass through the checkpoint to survive and proliferate [28, 29]. Therefore, Bach2 and Bcl6 modulate the two opposing cellular processes during the checkpoint (namely, apoptosis and survival), ensuring that the B cell repertoire is made up of cells carrying *V*_*H*_–DJ_*H*_ rearrangements.

**Figure 3:**
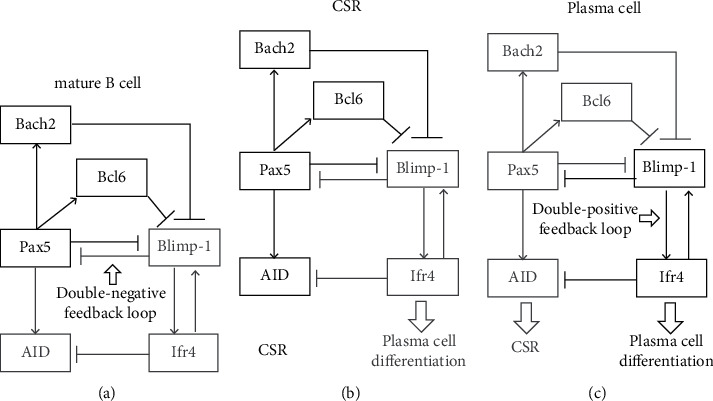
The transition of the core transcription factor regulatory network from CSR to plasma cell differentiation. (a) Pax5 and Blimp-1 form a double-negative feedback loop, in which Bach2, Bcl6, and Pax5 repress Blimp-1 to maintain the state of mature B cells. (b) Bach2 serves as an essential controller to determine the time window for AID expression and CSR completion by repressing Blimp-1. (c) The double-positive feedback loop between Blimp-1 and Ifr4 is activated to inhibit Pax5, Bach2, and AID, thereby promoting the differentiation to plasma cells. Black boxes and arrows represent activated genes and pathways, and gray represent surpressed genes and pathways.

During the germinal center (GCs) stage, B cells generally undergo two forms of transformation: Ag-activated B cells and terminally differentiated cells (plasma cells or memory B cells). After rapid clonal expansion, Ag-activated B cells undergo somatic hypermutation (SHM) and class switch recombination (CSR) to differentiate into antibody-secreting plasma cells or antibody-isotype memory B cells [30]. This differentiation process is regulated by many transcription factors, especially Pax5 and Blimp-1. Pax5 and Blimp-1, forming a double-negative loop, are recognized as a binary switch used to define either B cell (Pax5^high^/Blimp-1^low^) or plasma-cell (Pax5^low^/Blimp-1^high^) status [30]. At the early stage, Pax5, Bach2, and Bcl6 still maintain high expression levels. By directly binding to the encoding gene *Prdm1,* they repress the Blimp-1 expression to stabilize the B cell status (Figure 3(a)). Meanwhile, the high expression level of Bach2 helps to maintain the expression of Pax5 and inhibits GC-B cell apoptosis [30, 31]. This is completely opposite to the proapoptotic function of Bach2 in the checkpoint phase. It is demonstrated that B cells from Bach2-deficient mice have reduced cell cycle progression and increased apoptosis in response to BCR signaling. The *Bach2*^−/−^ B cell population show an elevated frequency of sub-G1 cells and early apoptotic cells compared with WT B cells [31]. This suggested that Bach2 is required to ensure the normal cell cycle and promote GC-B cell proliferation.

For SHM and CSR, Bach2 is an essential regulator and time controller. Muto et al. reported that genetic ablation of Bach2 in B cells results in a marked reduction in the progression of CSR and SHM and an increase of IgM production [32]. The Ag-driven differentiation of IgM plasma cells is intact but specific IgG antibodies are not induced in Bach2-deficient mice. This indicates impaired CSR under Bach2-deficient status. Nucleotide changes are also impaired in Bach2-deficient lymphoid cells, implying the critical role that Bach2 plays in SHM [32]. As discussed earlier, Bach2 supports the Pax5 expression and suppresses Blimp-1. The Bach2-mediated repression of Blimp-1 is essential for execution of CSR and plasma-cell differentiation [30]. It is well known that SHM and CSR critically depend on the enzyme activation-induced deaminase (AID) that is induced by Pax5 [8, 33]. During CSR, Bach2 expression gradually decreases and greatly reduces when CSR are successfully completed, while the expression of Blimp-1 and Irf4 gradually increases due to lack of Bach2 repression. Blimp-1, on one hand, downregulates the AID in a Pax5-inhibition manner to terminate the CSR, and, on the other hand, upregulates Irf4 (which in turn activates *Prdm1*) to form a double positive feedback loop to promote plasma-cell differentiation [7, 8, 34] (Figures 3(b) and 3(c)). Once the differentiation is completed, the increased Blimp-1 with the low levels of Pax5 and Bach2 maintains the state of plasma cells. Thus, it can be seen that the length for the Bach2 repression of Blimp-1 determines the time window for AID expression and CSR completion, which in turn controls the delay of GC-B cell differentiation (Figures 3(b) and 3(c)). This process is defined as “delay-driven diversity model of CSR” by Muto et al. [8, 30]. Supporting this theory, studies have shown that Bach2-deficient B cells more rapidly express Blimp-1 and then quickly differentiate into IgM plasma cells compared with wild-type B cells [30, 35]. The defect in CSR of Bach2-deficient B cells is rescued by the simultaneous ablation of *Prdm1*, because the Bach2/Blimp-1-double defect restores the AID expression [30].

Another important form of terminal differentiation is memory B cells, which also require large amounts of Bach2 for generation [36]. Studies showed that knockdown or genetic ablation of Bach2 in mice leads to the failure of memory B cell generation and predisposes the B cells towards plasma-cell differentiation without antigen stimulation [36, 37]. This suggests that Bach2 is necessary for the generation and development of memory B cells. However, the precise mechanisms by which Bach2 regulates the differentiation of memory B cells remain to be elucidated.

One characteristic of Th2 allergic response is the excess production of high-affinity IgE from B cells. Recent studies show that the IgE BCR has distinct signaling properties from the IgG1 [38, 39]. The generation of high-affinity IgE requires GC-B cells that express IgG1 to first undergo affinity maturation and differentiate into memory cells and subsequently switch to IgE upon allergen reexposure [40]. As mentioned above, Bach2 is essential for the development of memory B cells [36, 37] and regulates SHM and CSR during GC reactions [8], which are all critical for IgE generation. Therefore, it is logical to speculate that Bach2 has an important role in regulating the production of high-affinity IgE in B cells and thus contributes to Th2 allergic response. This remains unelucidated and requires further investigation.

### 3.3. The Effect of Bach2 on Tfh Cell Differentiation and Function

Tfh cells, a distinct CD4 T-cell subset, usually locate in B cell follicles in the secondary lymphoid organs and provide help for GC B cell reactions and GC formation [41–44]. Tfh cells primarily modulate affinity maturation and isotype of antibody produced by B cells and promote long-lived plasma-cell differentiation and memory B cells generation [43].

The Bach2 expression is low in Tfh cells, and its overexpression results in a rapid loss of the Tfh cell phenotype and subsequent abolishment of GC response [18, 19]. Reciprocally, when Bach2 is conditionally deleted in T cells, there are spontaneous accumulation of Tfh cells and formation of GCs [45]. It is evident that Tfh cells are functionally dependent on low levels of Bach2 to maintain their phenotype and prevent the dedifferentiation of Tfh cells into non-Tfh effector cells [18]. This notion is further supported by the observation that Bach2-deficient Tfh cells are driven to the IL-4-secreting subset, leading to increased generation of IgE^+^ B cells [45]. It also implicates the critical role of Bach2 in humoral autoimmunity, partially by inhibiting the generation of pathogenic Tfh cells. On the other hand, Bach2 overexpression does not lead to increased apoptosis, reduced proliferation, or egress out of lymph nodes due to loss of Tfh cells, suggesting that Bach2 does not regulate the proliferation, survival, or migration of Tfh cells [18].

Instead, Bach2 regulates Tfh cell generation and function. Lahmann et al. report that the Bach2 overexpression reduces Bcl6, one of the key transcription factors involved in Tfh cell differentiation [18]. Reciprocally, acute deletion of Bcl6 in Tfh cells increases the Bach2 expression [45]. As aforementioned, Bach2 dimerizes with Batf through its bZip domain to compete the Batf–Irf4 complex for binding to the *Bcl6* promoter and suppress the Bcl6 expression [18]. The transcriptional repression function of Bach2 is dependent on its bZip region because deletion of the bZip region resulted in unrepressed Tfh cell differentiation, even under Bach2 overexpression conditions [19]. Although Bcl6 and Blimp-1 are reciprocal antagonists in Tfh cells, Bach2 does not indirectly regulate Bcl6 via suppressing Blimp-1. In contrast, Bach2 in Tfh cells is downregulated to prevent direct inhibition of Bcl6 [18]. Interestingly, in Tfh cells differentiated from Bach2-deficient T cells, the expression of both Bcl6 and Blimp-1 is increased [19]. This indicates that these Tfh cells may be resistant to Blimp-1-mediated suppression, and there are other unelucidated mechanisms in the Bach2-deficident condition that require further investigation.

**Figure 4:**
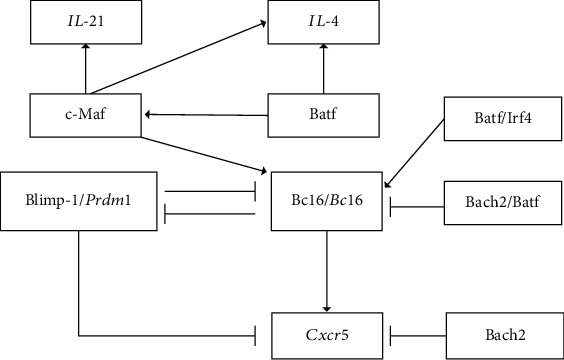
The core transcription factor regulatory network of Tfh cell differentiation in mice.

Bach2 also represses several other Tfh cell-related genes, including CXCR5, c-Maf, coinhibitory cell surface receptor TIGIT, and IL-21 [18, 19, 45]. c-Maf is a positive regulator of Tfh cell differentiation, and it induces the expression of various Tfh-related genes such as CXCR5 and IL-21 [46, 47]. In Bach2-deficient Tfh cells, both c-Maf and CXCR5 gene expressions are increased [45]. The deletion of Bach2 upregulates CXCR5 expression before the induction of achaete-scute homologue 2 (Ascl2), which is considered as the initiation factor of Tfh cell differentiation and direct regulator of CXCR5 [19, 48]. Bach2 binds to the regulatory element which is 36 kb upstream of the transcription start site of CXCR5 locus and suppresses CXCR5 upregulation [19]. Interestingly, Bach2 deficiency is not sufficient for the CXCR5 expression, and the CXCR5 overexpression does not revert the Bach2-mediated suppression of Tfh cell differentiation [19]. This suggests that Bach2 may be necessary for CXCR5 regulation and, hence, Tfh cell differentiation; however, there are other genes and mechanisms involved in this process which remain unelucidated. In conclusion, Bach2 is a negative regulator for Tfh cells, and it directly represses key Tfh genes, including Bcl6 and CXCR5 (Figure 4). Bach2 is required to regulate the secretion of IL-4 from Tfh cells and the generation of IgE+ B cells, which highlights its role in the Th2 immune response. However, the underlying mechanisms remain to be elucidated.

### 3.4. The Effect of Bach2 on Treg Cell Differentiation and Function

Treg cells are considered as an anti-inflammation CD4^+^ T-cell subset and are well known for their immunosuppressive function. Therefore, they primarily serve as negative regulators to suppress the Th2 cell-mediated immune reaction and inflammation.

Bach2 is highly expressed in thymic Treg (tTreg) cells, while different subsets of Treg cells in peripheral tissues have both low and high Bach2 expression under noninflammatory conditions [49]. In tTreg and resting Treg (rTreg) cells, highly expressed Bach2 inhibits IL-2 receptor signal and TCR signal to maintain their resting and inactive state, thus preventing rTreg differentiation into effector Treg (eTreg) cells and promoting the long-term survival of tTreg cell population [49–51]. In eTreg cells, the Bach2 expression becomes low, while the maturation marker kills the cell lectin-like receptor subfamily G1 (KLRG1), and Blimp-1 shows high expression [51]. This is consistent with the observation that the Bach2 expression is downregulated in a subset of peripheral Treg (pTreg) cells in response to inflammation [49]. A low Bach2 expression allows eTreg cells to effectively respond to specific antigens but may also lead to hyperfunction of eTreg cells under the condition of dysregulated immune homeostasis. In Bach2-deficient Treg cells, genes involved in eTreg differentiation are upregulated, whereas genes associated with the naïve Treg state are downregulated [51]. This further supports that Bach2 is required in naïve and early Treg cells to prevent premature activation and differentiation into eTreg cells, thus maintaining the homeostasis of Treg cell subsets.

In addition, Bach2 is also crucial for the development of Treg cells because it is required to induce the Foxp3 expression. In Bach2-deficient mice, Foxp3^+^ tTreg cells are significantly reduced, and transcription factors that promote Treg differentiation such as GATA1 and Foxo1 are also substantially reduced [10]. The conventional CD4^+^ T cells isolated from these Bach2-deficient mice also display propound impairments of the Foxp3 expression and generation of Foxp3^+^ pTreg cells [10]. For example, when stimulated under the pTreg condition with IL-2 and IL-2 plus TGF*β*1, Bach2-deficient CD4^+^ T cells preferentially develop into non-Foxp3 T effector cells such as Th1, Th2, and Th17 cells [5], suggesting that pTreg differentiation is impaired in the absence of Bach2. Importantly, the defect of pTreg induction in Bach2-deficient CD4+ T cells can be rescued by Bach2 sufficiency [5].

The findings discussed above suggest that Bach2 is indispensable in the development of Treg cells, but the mechanism by which Bach2 regulates Treg cell development has not been fully elucidated. The differentiation of pTreg is unrelated to the effects of Bach2 on restraining expression of proinflammatory mediators such as IFN-*γ* and IL-4 or transcription factors such as GATA3 [51]. Instead, pTreg cells require Bach2 to counteract TCR-induced transcription activities and to induce the Foxp3 expression. Studies indicate that Bach2 directs the differentiation of both pTreg and tTreg cells in the opposite direction of Irf4, which mediates TCR-dependent transcription in a dose-dependent fashion, and thus is critical in the differentiation of tTreg cells into fully functional eTreg cells [51, 52]. It is reported that the additional loss of Irf4 by using a Bach2/Irf4 double knockout (KO) mouse model restores the Foxp3 expression of Bach2-deficient CD4^+^ T cells [51]. Moreover, Irf4 KO mice exhibit impaired eTreg cell generation and lack of eTreg markers, including TIGIT and KLRG1, and additional loss of Bach2 in the Irf4-deficient Treg cells rescues the expression of these eTreg markers [51]. Hence, Bach2 and Irf4 act in an opposing manner to control the differentiation of tTreg and pTreg cells in response to TCR signals. Interestingly, the Irf4-dependent block of pTreg differentiation in the absence of Bach2 is independent of the Foxp3 activity [51]. Instead, Bach2 competes with Irf4-recruiting AP-1 complex binding to target genes and counteracts the DNA-binding activity of Irf4 to limit chromatin accessibility [49, 51]. This restrains the activation of transcription post-Irf4 TCR signals and thereby attenuates premature eTreg cell differentiation.

## 4. Bach2 and Th2 Immune Response/Th2 Inflammation

Bach2 plays a critical role in the pathogenesis of Th2-mediated inflammatory diseases by regulating the differentiation of Th2-related immune cells. It is identified that single-nucleotide polymorphisms (rs1847472 and rs10455168) of the human Bach2 gene loci are correlated with asthma risks [39, 53]. Moreover, recent studies have advanced the understanding that Th2-type inflammation and Th2 inflammatory responses are also found in autoimmune diseases such as multiple sclerosis and rheumatoid arthritis [54, 55]. Genome-wide association studies have identified various genomic susceptibility loci of Bach2 that are linked with multiple sclerosis and rheumatoid arthritis [56, 57].

### 4.1. Th2 Airway Inflammation

The role of Bach2 in Th2-type airway inflammation is first reported by Roychoudhuri et al. in a Bach2 KO mouse model, showing that Bach2 KO mice develop severe lung inflammation with extensive perivascular and alveolar infiltration of lymphocytes, macrophages, and eosinophils [5]. This phenotype is highly reminiscent to the Th2 type immunity-driven chronic pulmonary inflammation. The number of CD4^+^ T cells in the lungs of these mice is markedly increased, accompanied by elevated levels of IL-4 and IL-13, while Treg cells skew towards Th2-like cells with increasing expression of *gata3* and Th2 cytokine genes [5, 10].

Furthermore, Kuwahara et al. report that mice with T-cell-specific deletion of Bach2 also develop Th2-type airway inflammation, such as goblet cell metaplasia, mucus hyperproduction, airway hyperresponsiveness and enhanced infiltration of eosinophils and lymphocytes in bronchoalveolar lavage fluid, and the peribronchiolar regions [6]. The number of IL-5- and IL-13-producing CD4^+^ T cells is also elevated in the lungs of these mice, accompanied with increased production of Th2 cytokines. However, unlike the Bach2-deficient mice, the mice with T-cell-specific deletion of Bach2 do not appear to develop Th2 inflammation in other organs such as kidneys, stomach, small and large intestines, liver, or pancreas [6]. Kuwahara et al. attribute this unique phenomenon to IL-33 and its receptor (IL-33R*α*) because the expression of *Il33* mRNA and the number of IL-33R*α*^+^CD4^+^ T cells are only increased in the lungs, not in other organs [6]. The IL-33R*α*^+^CD4^+^ T cells with Bach2 deficiency exhibit characteristic features of aberrant Th2 cells and release large amounts of Th2 cytokines [9, 58].

In addition, mice with global Bach2 null develop more severe and fatal inflammation than those with T-cell-specific Bach2 null. Mice with global Bach2 deficiency exhibit the age-dependent increase in morbidity and mortality starting from 8 weeks of age. Some mice with global Bach2 deficiency develop moderate respiratory distress as early as 4 months, and by 7–8 months, almost all global Bach2 KO mice display severe and often lethal respiratory distress [10]. In contrast, this level of fatality is not observed in T-cell-specific Bach2 KO mice [6]. The underlying cause of these two phenotypes between mice with global Bach2 deficiency and those T-cell-specific Bach2 null remains to be investigated, but it is likely to be multifactorial. Mice with global Bach2 null not only lack Bach2 in T cells but also in B cells. It is reported that in global Bach2 null mice, pulmonary veins are mainly encircled by dense cuffs of plasma cells, and atypical plasma cells with marked cytomegaly and karyomegaly could be found in the perivascular cuffs of the most severely affected mice [10]. In this regard, Bach2-deficient B cells may synergize with Bach2-deficient T cells to amplify Th2 airway inflammation, leading to lethal lung inflammation.

### 4.2. Th2 Inflammation in Autoimmunity

Autoimmune diseases such as multiple sclerosis and rheumatoid arthritis are traditionally recognized as Th1-mediated immune responses, but emerging evidence suggests that Th2-mediated inflammation is also involved. For example, CD4^+^ T cells with Th2 signatures accumulate in pattern II plaques from autopsy cerebral tissue of patients with multiple sclerosis [59]. CXCR3^+^ Th2 cells are also found in synovial fluid in addition to Th1 cells in patients with rheumatoid arthritis [55]. Moreover, it is hypothesized that IL-4 and Th2 immune responses are involved in the coronavirus disease of 2019- (COVID-19-) associated male infertility [60].

The imbalance of immune tolerance mediated by Treg cells and the production of autoantibodies are considered to be the main causes of autoimmunity. As aforementioned, Bach2 plays a key role in regulating Treg cells. Zhang et al. report that mice with Treg-cell-specific Bach2 deficiency develop the late onset systemic autoimmunity in multiple organs, manifested by increasing CD4^+^ T cells and elevating the production of proinflammatory cytokines and autoantibodies [50]. This phenotype is similar to that found in mice with the global Bach2 null by Roychoudhuri et al. [5]. The autoimmunity suggests the loss of pTreg development due to Bach2 deficiency and resultant Th2 type inflammation. Similarly, T-cell-specific Bach2 KO mice also develop autoantibodies, suggesting the coexistence of Th2-type inflammation and autoimmunity [45]. This is possible because Bach2 regulates cells involved in both Th2 immune responses and autoimmunity, such as B cells, Tfh cells, and Treg cells, even though the activities of these cells are distinct. This also underlies that the functions of Bach2 are multifaceted. Given the critical role of Bach2 in both Th2 type inflammation and autoimmunity, Bach2 may function differentially in Th2 type inflammation within an autoimmune disease. Further investigations will help to understand the complex role of Bach2 in Th2 type inflammation and autoimmunity.

## 5. Conclusion

In this review, we have demonstrated that Bach2 is a critical immune-regulating transcription factor in Th2, Tfh, Treg, and B cells and plays an important role in Th2 immune response. The function of Bach2 is distinct in each Th2-related cell type, and Bach2 regulates differentiation and development of these cells. Bach2 serves as a negative regulator of Th2 and Tfh cells, while it controls differentiation of Treg cells originated from thymus and peripheral tissues in an antagonistic manner. Bach2 is also involved in every stage of B cell development with distinct functions. Bach2 is emerging as a fundamentally critical molecular “switch” that can restrain the Th2-driven differentiation of both B cell lineages and T-cell lineages and in turn constrains excessive Th2 functional activities and inhibits the Th2-mediated pathogenesis. Nevertheless, the regulatory pathways of Bach2 still have many unknowns. First, little is known about the pathways that regulate the Bach2 expression in both T and B cells. Second, the molecular mechanisms that Bach2 regulates the differentiation and development of Treg and Tfh cells have not been fully elucidated. Thirdly, the regulatory pathways of Bach2 in the generation of memory T and B cells are still unclear. Further research and in-depth understanding will facilitate the development of new approaches to restrain pathological Th2 responses and explore new strategies for treatment.
